# The Role of Visual Perception in Reading Across Fonts and Similar Words in Children with Reading Disabilities

**DOI:** 10.1007/s44402-026-00094-4

**Published:** 2026-05-06

**Authors:** Marc Argilés, Cristina Rovira-Gay, Liat Gantz, Luis Pérez-Mañá, Bernat Sunyer-Grau, Joan Gispets

**Affiliations:** 1 Universitat Politècnica de Catalunya - BarcelonaTech (UPC), Campus Terrassa, Barcelona, Spain; 2 Centre for Sensors, Instruments and Systems Development (CD6), Universitat Politècnica de Catalunya - BarcelonaTech (UPC), Campus Terrassa, Barcelona, Spain; 3 Department of Optometry and Vision Science, Jerusalem Multidisciplinary College, Jerusalem, Israel; 4 Vision, Optometry and Heath - VOS Research Group, Universitat Politècnica de Catalunya - BarcelonaTech (UPC), Barcelona, Spain

**Keywords:** Learning, Reading disabilities, TVPS, Visual perception

## Abstract

**Purpose:**

This study examined associations between visual perceptual skills and reading performance in children aged 6–12 years, focusing on reading similar words and words in different fonts.

**Methods:**

Forty-six children (mean age 8.3 ± 0.9 years; 24 and 22 with and without reading disabilities, respectively) were assessed. Visual discrimination, visual memory and form constancy were examined with the TVPS-3 (Test of Visual Perceptual Skills, 3rd version). Reading speed was measured using the TALEC test (Test d’Anàlisi de la Lectoescriptura en Català). Additionally, the reading speed and accuracy of reading lists of similar words, as well as words presented in six different fonts, were recorded.

**Results:**

Children with reading disabilities scored significantly lower for visual discrimination, visual memory and form constancy (all *p* < 0.01) and showed slower reading speed and higher error rates for both word list tasks (all *p* < 0.01). In this group, visual discrimination and visual memory correlated significantly with reading similar words, while visual memory correlated significantly with reading different fonts. In children without reading disabilities, form constancy correlated strongly with both tasks and TALEC performance (*p* = 0.002).

**Conclusion:**

Visual perceptual skills influence reading performance differently in children with and without reading disabilities. While these skills are important to assess, low scores on visual perception tests such as the TVPS-3 do not necessarily predict reading between similar words and different fonts, highlighting the need for a more comprehensive evaluation in clinical optometry.

Key Points

- Children with reading disabilities demonstrated significantly lower performance in visual discrimination, visual memory and form constancy compared with children without reading disabilities.
- The reading speed and accuracy of children with reading disabilities were significantly reduced, particularly when processing visually similar words or varying fonts, compared with children without reading disabilities.
- No associations were found between visual perception skills, such as visual discrimination, visual constancy and visual memory for reading similar words and different fonts, indicating that low scores with the Test of Visual Perceptual Skills, 3rd version, should not be interpreted as affecting reading performance directly.

## Introduction

Reading is a fundamental skill that underpins a child’s academic success and cognitive development. Reading proficiency involves not only the ability to decode written text, but also to comprehend and engage meaningfully with its content [1]. In recent years, research has highlighted the complex and multifaceted nature of visual perception in reading [2], emphasising the critical role of underlying cognitive processes in reading acquisition and fluency [3].

Developmental dyslexia (DD) is a heterogeneous neurodevelopmental disorder characterised by multiple cognitive profiles rather than a single underlying deficit [4, 5]. Reading difficulties in dyslexia have been associated with impairments in word perception [6], visual attention [7] and phonological deficits [2, 8, 9]. In particular, visual processing impairments have been described within the magnocellular theory, which posits that dysfunction in the magnocellular visual pathway compromises the processing of rapid visual information, thereby affecting eye movement control, visual attention and temporal integration mechanisms essential for fluent reading [10, 11]. Additionally, DD shows high rates of comorbidity with other neurodevelopmental conditions, most notably attention-deficit/hyperactivity disorder (ADHD), suggesting partially shared cognitive and genetic risk factors that contribute to variability in symptom presentation and severity [12, 13]. Together, these findings support a multifactorial and multidimensional view of DD, emphasising the interaction of phonological, visual and attentional mechanisms in its aetiology rather than a unitary causal pathway. Also, deficits in visual perception have been associated with DD [14]. These findings underscore the importance of visual perceptual skills for reading accuracy [15] and comprehension [16].

Moreover, visual perception difficulties in children can negatively impact multiple learning-related abilities, including spelling, writing and numeracy [17], ultimately leading to lower academic achievement [18]. They have also been linked with poorer handwriting legibility [19], reduced social skills [20], diminished self-esteem and a lower overall quality of life [17]. For instance, children with visual discrimination problems may confuse visually similar words, such as left/lift, wet/went or turn/torn [17]. Despite these associations, the precise mechanisms through which visual perception influences reading performance remain insufficiently understood. Certain genetic disorders, such as Turner syndrome, have been linked to reading impairments, partly due to deficits in form constancy [21]. Children with such disabilities may struggle to distinguish visually similar letters, particularly when presented in different fonts or orientations.

Visual perception refers to the ability to interpret and process visual information from the environment, enabling recognition of shapes, patterns and spatial relationships [22]. Visual perception is composed of several subskills, which can be assessed using standardised clinical tests such as the Test of Visual Perceptual Skills (TVPS) [23] and the Motor-Free Visual Perception Test [24]. The TVPS is widely used for diagnosis in children aged 4–18 years and measures seven subtypes of visual perception: visual discrimination, visual memory, spatial relationships, form constancy, sequential memory, figure-ground and visual closure [25, 26]. These subskills play distinct roles in reading. For example, visual discrimination enables differentiation of letters and words [27], while sequential memory supports word recognition and comprehension [28]. Form constancy allows recognition of letters regardless of font or size and has been shown to predict reading success in logographic scripts such as Chinese [27].

Research findings on the effects of different fonts on individuals with reading disabilities are mixed. While some studies suggested that font type can affect readability [29], others reported no significant effects [30, 31]. Similarly, the association between visual perception skills and letter recognition is debated, especially when linguistic abilities are controlled for [32]. Other factors, such as visual crowding [33], attentional difficulties [34] and text characteristics [35], can further influence reading outcomes.

Despite these findings, few studies have examined how specific visual perceptual subskills relate to reading performance in children with and without reading difficulties or disabilities, particularly in the context of different font types and reading similar words. For example, in clinical optometric practice, when visual discrimination skills are low, individuals may have difficulty differentiating between visually similar words (e.g., left/lift). Further, when form constancy is weak, the recognition of letters and words across different fonts and sizes may be impaired, and when visual memory is limited, retention of orthographic patterns necessary for fluent reading may be compromised.

However, while these associations are widely considered in clinical practice, they have not been verified systematically through empirical research. To our knowledge, no prior study has examined directly whether these specific visual perceptual skills, evaluated with the TVPS-3 test, are differentially associated with reading speed, reading visually similar words and reading with different fonts in children with and without reading disabilities. The present study tested these clinical assumptions by assessing visual discrimination, visual memory and form constancy in children aged 6–12 years with and without reading disabilities, and by examining their association with reading speed and accuracy across standard tasks as well as using lists of visually similar words and words presented in multiple fonts.

## Methods

### Participants and Inclusion Criteria

The study included 46 children (mean age ± standard deviation: 8.3 ± 0.9 years), comprising 24 boys (52.17%) and 22 girls (47.83%), recruited from two private primary schools in Girona, Spain. Information on whether a participant had a diagnosed reading disability was obtained through a questionnaire completed by their legal guardians. Additionally, the psychology department at each school confirmed that all participants had age-appropriate intelligence based on the WISC (Wechsler Intelligence Scale for Children) test and a diagnosis of reading disabilities. In Catalonia, screening for reading difficulties is not mandated universally; typically, children are referred for psychoeducational evaluation when teachers observe persistent reading difficulties, and assessments are conducted by school psychologists or external specialists. Although a formal intelligence quotient (IQ) cutoff was not established within the present study, school psychologists reported that all participants had age-appropriate intellectual functioning based on prior assessments conducted as part of routine educational practice. Control children had no history of learning or developmental disorders, as confirmed by school records and parent report, and did not undergo a standardised cognitive assessment (e.g., WISC) within the framework of this study. All children were native Catalan speakers. Written informed consent was obtained from all legal guardians after they received a detailed explanation of the study’s nature and procedures. This study followed the tenets of the Declaration of Helsinki (1975, revised in Tokyo in 2013) and was approved by the UPC Institutional Review Board (approval number 22/365882).

The inclusion criteria were as follows: participants aged between 6 and 12 years, corrected visual acuity of 0.05 logMAR or better in both eyes at both distance and near, no history of ocular pathology or ocular surgery, absence of strabismus as determined by the cover test at 6 and 0.4 m, a near point of convergence <10 cm, with recovery using letters corresponding to a decimal visual acuity of 0.7 and normal monocular amplitude of accommodation for each participant’s age (based on Hofstetter [36],) measured in the right eye using the push-away method with an RAF rule.

### Measures

Testing was conducted by a trained optometrist, a native Catalan speaker, in a quiet room at the two participating schools during regular school hours. Prior to testing, inclusion criteria were verified through assessments of distance and near visual acuity and cover test, as well as near point of convergence and amplitude of accommodation tests. Following verification, reading comprehension and speed were evaluated using the following non-invasive measures, administered in random order for each participant:

(a) Analysis of Reading and Writing in Catalan with TALEC (Test d’Anàlisi de la Lectoescriptura en Català) to evaluate reading comprehension and speed. This test consists of four reading levels, with corresponding comprehension questions. Participants were presented with age-appropriate texts and instructed to read them aloud as quickly as possible. Reading speed (in seconds) was measured manually for each participant, from the beginning to the end of the passage. Reading speed was used as a variable to examine possible correlations with performance in visual perception skills and reading across different typefaces, without introducing diagnostic subgroups, in order to explore at a general level whether reading difficulties may be related to visual perception skills evaluated with the TVPS.

(b) Visual Discrimination, Visual Memory and Form Constancy subskills from the TVPS (3rd version) to assess participants’ visual perceptual abilities without requiring a motor response. This standardised, norm-referenced assessment measures visual perception across multiple domains in 4–18-year-olds. It provides age-based normative percentile scores for each subtest and for the overall performance, allowing for direct comparison with same-age peers. Results of these subskill tests (in percentiles) were compared with other study measures to assess associations.

(c) Word List with Different Fonts: This task was developed specifically for this study and consisted of 48 words presented in six different font types, represented by eight randomly assigned words (see Supplementary Information, Table S1: *List of words displayed across six font types*). For children younger than 9 years, a shortened version of 24 words was administered, while children aged 9 years and older completed the full set. Participants were instructed to read the words aloud as quickly and accurately as possible.

The word list was presented in six different fonts: Monotype Italics, Lucida Console, Bradley Hand, Mistral, Harrington and Calibri. These fonts were chosen to capture a range of typographic features that may influence reading performance, particularly the presence of serifs, the degree of stroke contrast and the potential for visual crowding. More descriptive and visual examples of the words used are shown in Supplementary Information, Table S2: *Examples of font characteristics* and Fig. S1: *Visual examples*. During the selection process, school teachers were consulted based on their experience and familiarity with fonts commonly used in educational materials. The teachers confirmed that the selected fonts closely resembled those typically found in school textbooks and academic reading materials, and were therefore considered appropriate for the purposes of this study. Serif fonts such as Monotype Italics and Harrington include small decorative strokes at the ends of letters; features often associated with more formal or traditional typefaces.

The fonts also differed in their degree of spatial crowding, which can impact word recognition and reading fluency [37, 38]. Monotype Italic, Bradley Hand and Calibri exhibit balanced spacing, similar to standard educational print fonts, whereas Mistral and Lucida Console are comparatively condensed. In contrast, Harrington features slightly extended proportions, which give it an elegant and decorative appearance [39]. By varying fonts across these dimensions systematically, the study simulated real-world reading conditions in which children may encounter diverse typographic styles. This variability allowed examination of whether specific visual perceptual skills (e.g., visual discrimination, form constancy and visual memory) are associated with children’s ability to adapt to different font characteristics during reading tasks.

(d) Two Word Lists with Similar Words: Two word lists in Catalan, developed specifically for this study, consisted of orthographically and phonologically similar words chosen with the support of the school teachers, who were asked to suggest words that are commonly encountered and frequently confused by children during reading. One list comprised one-syllable words, while the second list consisted of two-syllable words. The one-syllable word list was administered to children younger than 9 years of age, while the two-syllable word list was used for children aged 9 years or older. Both lists are provided in the Supplementary Information, Table S3: *Word lists of similar words*. Participants were instructed to read the words aloud as quickly and accurately as possible, while the reading time and the number of errors were recorded.

Within each list, words were deliberately chosen to be orthographically and phonologically similar (e.g., nas/pas/ras, cara/cama/casa) to increase the likelihood of visual confusion during rapid reading. This design aimed to challenge visual discrimination, an essential perceptual skill that helps readers distinguish between words with minimal differences in letters or structure. Additionally, letter position similarity was incorporated (e.g., pal/mal/tal, poma/goma/cama). These lists were constructed using frequent, age-appropriate vocabulary to minimise semantic unfamiliarity and isolate the visual-perceptual demands of the task. This design enabled the assessment of whether visual discrimination, visual memory and form constancy affect performance when processing highly similar word forms, a challenge frequently encountered in early reading and spelling development.

### Statistical Analysis

All data were recorded in spreadsheets. The Shapiro–Wilk test indicated a non-parametric distribution for the following variables: reading similar words in the group of children with reading disabilities (*p* = 0.02); reading different words in different fonts in children with and without reading disabilities (*p* = 0.008 and *p* = 0.02, respectively); visual discrimination in the group of children with reading disabilities (*p* < 0.001); visual memory in children with and without reading disabilities (*p* < 0.001 and *p* = 0.048, respectively); visual constancy in both groups (*p* < 0.001) and the TALEC test in the group of children with reading disabilities (*p* = 0.02). Descriptive statistics were reported as medians and interquartile ranges for the group of children with and without reading disabilities.

The non-parametric, unpaired Mann–Whitney test (*U* test) was used to compare the two groups. Rank-biserial correlation (*r*_rb_) was calculated for effect sizes. Spearman’s rank correlation (*ρ*) was applied to examine associations between TVPS subtests (visual discrimination, visual memory and form constancy) and both the TALEC test and the word list tasks (different fonts and similar words). To assess formally whether correlations differed between groups, Fisher’s *z* transformation was applied to compare independent correlation coefficients. The standard error for independent correlations under this approach was 0.32. To control for multiple comparisons, a Benjamini–Hochberg false discovery rate (FDR) correction was applied separately within each group (six comparisons per group). Using G*Power software (version 3.1.9.7; www.psychologie.hhu.de/arbeitsgruppen/allgemeine-psychologie-und-arbeitspsychologie/gpower) [40], the minimum sample size required to compare outcomes that were not normally distributed was calculated. Assuming a power (1-ß) of 95%, an alpha error probability of 5% (*α* = 0.05) and an effect size (*d*) of 1.67 to detect at least a 10% difference in the TVPS test with a standard deviation of 6 [41], the required sample size was determined to be 22 participants per group. A *p* value < 0.05 denotes statistical significance.

### Ethics Approval and Consent to Participate

All procedures performed in this study involving human participants were in accordance with the ethical standards of the Institutional Review Board of the Universitat Politècnica de Catalunya (UPC, approval number 22/365882) and with the 1964 Declaration of Helsinki and its later amendments. Written informed consent was obtained from the legal guardians of all participating children prior to their inclusion in the study.

## Results

This study included 46 participants, divided into two groups, i.e., with and without reading disabilities. The group of children with reading disabilities (*n* = 24) included 14 boys (58.33%) and 10 girls (41.67%), with a mean age of 8.3 ± 1.1 years. The group of children without reading disabilities (*n* = 22) included 10 boys (45.45%) and 12 girls (54.55%), with a mean age of 8.4 ± 0.6 years. The groups did not differ significantly in gender distribution (Fisher’s Test, *p* = 0.56) or age (Chi-Square Test, *χ*^2^ = 0.72, *p* = 0.79). Visual acuity, cover test at near and distance, NPC break and recovery and amplitude of accommodation were not statistically significant between groups (*p* > 0.05 for all variables; see Table 1).

**Table 1:** Descriptive information for optometric tests expressed as median and interquartile range (IQR: 25–75%).

	Entire cohort (*n* = 46)	Group of children with reading disabilities (*n* = 24)	Group of children without reading disabilities (*n* = 22)	*p* value
Visual acuity (logMAR)	0.00 (0.00, 0.00)	0.00 (0.00, 0.00)	0.00 (0.01, 0.00)	0.24
Cover test (Δ) at 6 m	0.00 (−4.00, 2.00)	0.00 (−2.50, 0.00)	0.00 (−4.50, 2.00)	0.92
Cover test (Δ) at 0.4 m	0.00 (−2.50, 0.00)	−4.00 (0.00, 0.25)	−2.00 (0.00, 0.00)	0.60
NPC-Break (cm)	5.00 (4.00, 6.00)	5.00 (5.00, 7.00)	4.75 (4.00, 6.00)	0.10
NPC-Recovery (cm)	5.25 (2.00, 6.00)	6.00 (3.00, 8.00)	5.00 (2.00, 7.00)	0.18
Amplitude of accommodation (D)	10.00 (9.09, 11.76)	10.00 (8.42, 11.76)	10.00 (9.41, 11.46)	0.81

Negative values in the cover test indicate exophoria.

*Δ* prism dioptres, *D* dioptres, *NPC* near point of convergence.

The outcomes of the reading tests are described in Table 2. Reading speed, measured in seconds with the TALEC test, was significantly higher for the group of children with reading disabilities (*U* = 112.00, *p* < 0.001, *r*_rb_ = 0.58). Percentile scores for all three visual perception skills were significantly lower in children with reading disabilities compared to those without reading disabilities. Specifically, significant group differences were found in visual discrimination (*U* = 85.0, *p* < 0.001, *r*_rb_ = 0.68), visual memory (*U* = 140.5, *p* = 0.006, *r*_rb_ = 0.47) and form constancy (*U* = 140.0, *p* = 0.005, *r*_rb_ = 0.47). Reading speed with similar words was significantly higher in the group of children with reading disabilities (*U* = 115.0, *p* < 0.001, *r*_rb_ = 0.56), with more errors (*U* = 106.5, *p* < 0.001, *r*_rb_ = 0.60). Reading with different fonts was statistically different between groups, with longer reading times in the group of children with reading disabilities (*U* = 127.0, *p* = 0.002, *r*_rb_ = 0.52) and more errors (*U* = 89.5, *p* < 0.001, *r*_rb_ = 0.66).

**Table 2:** Outcome measures of the study tests presented as median and interquartile range (IQR: 25–75%).

		Group of children with reading disabilities (*n* = 24)	Group of children without reading disabilities (*n* = 22)	*p* value
TALEC (Test d’Anàlisi de la Lectoescriptura en Català)	Time (seconds)	100.00 (72.25, 169.3)	65.50 (47.75, 100.00)	<0.001*
Visual discrimination	Percentile	12.50 (5.00, 25.00)	50.00 (25.00, 77.25)	<0.001*
Visual memory	Percentile	9.00 (1.00, 25.00)	25.00 (9.00, 53.25)	0.006*
Form constancy	Percentile	5.00 (1.25, 14.25)	16.00 (8.00, 66.00)	0.005*
Word list of similar words	Time (seconds)	73.50 (47.25, 87.50)	50.00 (39.50, 59.50)	<0.001*
	Errors	4.00 (2.00, 7.00)	1.00 (0.00, 2.00)	<0.001*
Word list of different fonts	Time (seconds)	66.00 (43.50, 111.8)	40.00 (34.25, 57.75)	0.002*
	Errors	4.00 (3.00, 7.75)	1.00 (0.00, 3.00)	<0.001*

Significant *p* values are highlighted with *.

In the group of children with reading disabilities, a significant negative correlation was observed between visual discrimination and reading speed for similar words (*ρ* = −0.48, *p* = 0.02), whereas no significant correlation was found in the group of children without reading disabilities (*ρ* = −0.18, *p* = 0.40). Visual memory demonstrated a significant negative correlation with reading speed for similar words in the group of children with reading disabilities (*ρ* = −0.48, *p* = 0.02), but not in the group of children without reading disabilities (*ρ* = −0.28, *p* = 0.21). A different trend was observed for form constancy: no significant correlation was found in the group of children with reading disability (*ρ* = −0.03, *p* = 0.89), whereas a significant negative correlation was present in the group of children without reading disabilities (*ρ* = −0.49, *p* = 0.02). Fisher’s *z* comparisons indicated that these differences were not statistically significant (all *p* > 0.05, Table 3). Adjusted *p* values after Benjamini–Hochberg FDR correction are reported alongside the original values in Table 3.

**Table 3:** Spearman correlation values (*ρ*) between visual perception skills and reading similar words and reading different fonts, both in speed (seconds) and errors.

	Velocity	Group of children with reading disabilities (*n* = 24)	Group of children without reading disabilities (*n* = 22)	Fisher’s *z* (*p* value)
Visual discrimination	Word list similar words Word list different fonts	*ρ* = −0.48, *p* = 0.02* [*p* = 0.04]* *ρ* = −0.41, *p* = 0.045 [*p* = 0.07]	ρ = −0.18, *p* = 0.40 [*p* = 0.40] *ρ* = −0.35, *p* = 0.11 [*p* = 0.21]	*z* = −1.08, *p* = 0.28 *z* = −0.31, *p* = 0.76
Visual memory	Word list similar words Word list different fonts	*ρ* = −0.48, *p* = 0.02* [*p* = 0.04]* *ρ* = −0.47, *p* = 0.02* [*p* = 0.04]*	*ρ* = −0.28, *p* = 0.21 [*p* = 0.29] *ρ* = −0.26, *p* = 0.24 [*p* = 0.29]	*z* = −0.89, *p* = 0.37 *z* = −0.87, *p* = 0.39
Form constancy	Word list similar words Word list different fonts	*ρ* = 0.03, *p* = 0.89 [*p* = 0.89] *ρ* = −0.10, *p* = 0.62 [*p* = 0.74]	*ρ* = −0.49, *p* = 0.02* [*p* = 0.05] *ρ* = −0.54, *p* = 0.008* [*p* = 0.048]*	*z* = 1.35, *p* = 0.18 *z* = 1.44, *p* = 0.15

*p* values after Benjamini–Hochberg correction are shown in brackets.

Significant *p* values are highlighted with *.

**Fig. 1:**
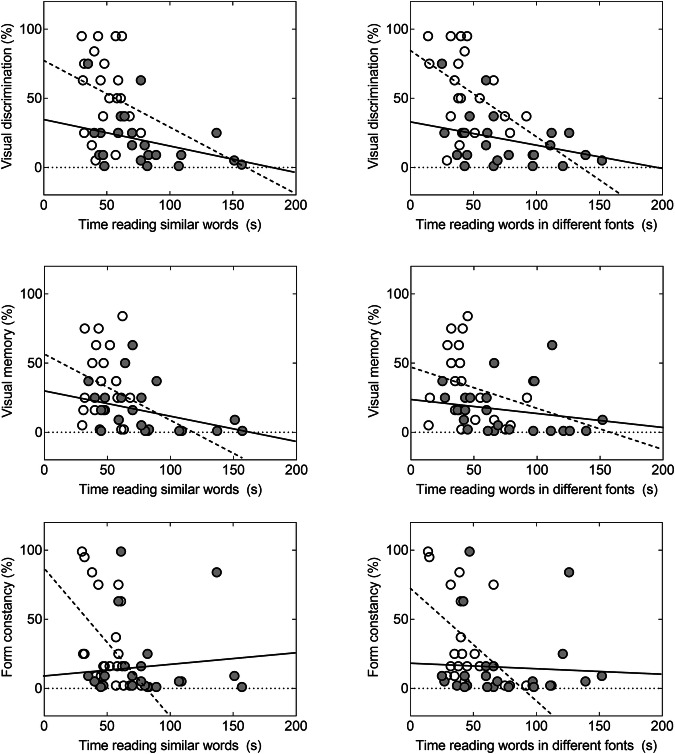
Spearman correlations (*ρ*) between the percentiles measured using the Test of Visual Perceptual Skills (TVPS) subtests (visual discrimination, visual memory and form constancy) and reading speed of with similar words (left panes) and different fonts (right panes). Grey-filled and open circles represent the groups of children with and without reading disabilities, respectively. Correlation lines are shown in solid black for the group of children with reading disabilities and dashed lines for the group of children without disabilities.

When reading words were presented in different fonts, a significant negative correlation was observed in the group of children with reading disabilities, indicating that the lower the visual discrimination, the greater the reading time (*ρ* = −0.41, *p* = 0.045). A significant correlation was not observed in the group of children without reading disabilities (*ρ* = −0.35, *p* = 0.11). Visual memory was significantly correlated with reading performance in the group of children with reading disabilities (*ρ* = −0.47, *p* = 0.02), but not in the group of children without reading disabilities (*ρ* = −0.26, *p* = 0.24). In contrast, form constancy was not significantly correlated with reading performance in the group of children with reading disabilities (*ρ* = −0.10, *p* = 0.62), whereas a strong negative correlation was observed in the group of children without reading disabilities (*ρ* = −0.54, *p* = 0.008). Fisher’s *z* comparisons indicated that these differences were not statistically significant (all *p* > 0.05). Table 3 summarises all the correlations between findings, while Fig. 1 shows the correlation plots for each visual perception skill and reading performance. After applying the Benjamini–Hochberg FDR correction within each group, most of the previously significant correlations in the reading disabilities group remained significant, with the exception of the association between visual discrimination and reading in different fonts. In the group without reading disabilities, only the correlation between form constancy and reading in different fonts remained statistically significant after correction, whereas the remaining associations did not survive adjustment.

No significant correlations were found between reading speed (assessed with the TALEC test) and visual discrimination (*ρ* = −0.30, *p* = 0.15), visual memory (*ρ* = −0.30, *p* = 0.14) or form constancy (*ρ* = −0.02, *p* = 0.91) in the group of children with reading disabilities. Similar trends were found in the group of children without reading disabilities between the TALEC test and visual discrimination (*ρ* = −0.39, *p* = 0.07) and the TALEC test and visual memory (*ρ* = −0.17, *p* = 0.43). Conversely, a strong correlation was found with form constancy (*ρ* = −0.61, *p* = 0.002).

A multiple regression analysis was conducted to predict reading performance based on visual discrimination, visual memory and form constancy. The overall model was significant, *F* (3,45) = 6.69, *p* < 0.01 and explained 32.3% of the variance in reading performance (*R*^2^ = 0.32, adjusted *R*^2^ = 0.28). Visual discrimination (*β* = −0.41, *p* = 0.008) was a significant predictor, while visual memory (*β* = −0.17, *p* = 0.21) and form constancy (*β* = −0.14, *p* = 0.34) were not significant predictors.

## Discussion

This study examined associations between visual discrimination, form constancy and visual memory with reading performance in children aged 6–12 years, comparing children with reading disabilities to their typically developing peers. These visual perceptual skills are often considered important in clinical practice, for example, for distinguishing visually similar words, recognising letters and words across different fonts and retaining orthographic patterns, but their relationship has not been examined systematically.

### Visual Perception Between Groups

Research has shown deficits in visual perception abilities in dyslexia [42], including auditory mechanisms [43], highlighting the heterogeneity within this population. Visual perception has also been linked to reading performance and the local and global perception of features [44]. Together, these studies demonstrate an association between visual perception and reading difficulties. However, in clinical practice, formal testing is not commonly used; rather, more ecologically valid assessments, such as the TVPS-3, are typically employed.

The group of children with reading disabilities presented clear weaknesses in visual discrimination (*p* < 0.001), visual memory (*p* = 0.006) and form constancy (*p* = 0.005) as measured by the TVPS-3. Research suggests that poorer readers may present a specific deficit in visual processing, while holistic processing remains relatively preserved or may even be enhanced as a compensatory strategy [45]. The association of visual perception between groups is consistent with Crawford and Dewey [46]. In the present study, children with reading disabilities demonstrated substantially lower percentile ranks and corresponding scaled scores across several visual-perceptual skills. For instance, the median percentile for visual discrimination in the reading disability group was 12.5, compared with 50 for the group of children without reading disabilities. Given that a 9-point percentile difference approximates to a 1-point scaled score difference in the TVPS-3 conversion system [23], this reflects a clinically meaningful gap of approximately 4–5 scaled score points.

Visual memory and form constancy also showed marked disparities. The median percentiles for the reading disability group were 9.0 (visual memory) and 5.0 (form constancy), compared to 25.0 and 16.0, respectively, in the group without reading disabilities. However, the very low median percentile observed for form constancy in the reading disability group suggests a possible restriction-of-range effect. Such floor-level performance may attenuate correlations, potentially obscuring the strength of the relationship between form constancy and reading within this group.

The regression analysis further highlights the specific role of visual-perceptual skills in reading performance. While the overall model was significant, explaining 32.3% of the variance in reading outcomes, only visual discrimination emerged as a significant predictor (*p* = 0.008). These results suggest that the results of this subtest in TVPS-3 may have a more direct impact on reading ability than visual memory and form constancy.

### Visual Perception in Reading Across Fonts and Similar Words

The selection of fonts in the present study was grounded in teachers’ expertise, ensuring that the typefaces reflected those typically encountered by students in everyday academic contexts. Nevertheless, beyond ecological validity, it is important to consider how specific typographic features may differentially influence reading performance. Arditi and Cho found that the presence or absence of fonts with serifs had no meaningful effect on adult reading speed, with only a very slight legibility advantage explained by increased letter spacing rather than the serifs themselves [47]. By contrast, fonts such as Lucida Console, Bradley Hand, Mistral and Calibri are sans-serif fonts, characterised by simpler, cleaner letterforms that are generally regarded as more legible in digital environments and at lower resolutions [27]. The variety of fonts differs in the degree of thickness contrast within letter strokes. For example, fonts such as Mistral and Harrington display high stroke contrast, providing visually decorative forms that may hinder rapid or fluent reading, particularly at smaller print sizes or for early readers. Monotype Italics demonstrates moderate stroke contrast, while Lucida Console, Bradley Hand and Calibri exhibit low stroke contrast, yielding more uniform and potentially easier-to-read letterforms. Such low-contrast fonts are typically recommended for functional texts because of their consistency and clarity [37]. Additionally, altering different fonts can change the spacing between letters and lines, which affects crowding. One study confirmed the relationship between crowding and reading performance [48].

In the present investigation, distinct correlation patterns emerged between visual perceptual skills and reading performance (i.e., reading similar words and words presented in different fonts) across groups. Specifically, in the group of children with reading disabilities, visual discrimination and visual memory were significantly associated with reading time in both similar-word and different-font conditions, whereas form constancy was not. In contrast, in the group without reading disabilities, form constancy showed significant correlations with reading performance, while visual discrimination and visual memory did not reach statistical significance.

Despite these differences in the within-group associations, Fisher’s *z* comparisons indicated that the strength of the correlations did not differ significantly between groups (all *p* > 0.05). Therefore, although the pattern of correlations varied descriptively across groups, it cannot be concluded that reduced visual-perceptual skills, such as visual discrimination, form constancy and visual memory, directly explain reduced performance in reading similar words or reading in different fonts. Although statistically significant differences in reading performance were observed between groups for both similar words (*p* < 0.001) and different fonts (*p* < 0.001), clinicians in optometric practice should be aware that a more comprehensive evaluation is necessary. Factors such as phonological processing, cognitive abilities and linguistic skills also contribute to reading performance and should not be assumed to be linked directly with these visual-perceptual skills.

### Limitations

As the aim was to examine, for the first time, the clinical assumptions regarding visual perception skills in reading across different fonts, this study was designed to reflect real-world clinical conditions. In routine clinical or school-based practice, comprehensive assessments of IQ, phonological processing and attentional abilities are not always available when evaluating children with reading difficulties. The goal here was to investigate whether visual perceptual skills, as measured by the TVPS, exhibit meaningful associations with reading performance using similar words and different fonts under ecologically valid conditions between students with and without reading disabilities. However, to maintain evidence-based research, certain limitations must be recognised.

First, factors such as IQ [49], emotional [50] and socioeconomic status [51] are known to influence reading performance in children aged 6–12 years. In the present study, both participating schools were private institutions from the same metropolitan area, and the school psychologists reported that all participants had age-appropriate IQs. However, IQ, emotional status and socioeconomic background were not assessed directly, which limits the ability to account for their potential influence on the findings. Moreover, as participants were recruited exclusively from private schools, the sample may not be representative of the general population, particularly students attending public schools.

Additionally, performance on the TVPS-3 may be influenced by domain-general cognitive factors such as sustained attention, working memory and executive functioning. Given the well-documented comorbidity between dyslexia and ADHD [12], it is possible that attentional factors partially mediated the observed associations between visual perceptual skills and reading. Future studies incorporating direct measures of attention and executive functioning would help disentangle specific visual-perceptual contributions from broader cognitive influences.

A further limitation is that the study did not differentiate between subtypes of reading disabilities or dyslexia, as well as the types of errors in reading (visual or phonological). Learning disabilities can manifest in multiple domains, such as reading, writing, mathematics and attention among children aged 6–12 years [52], and the consequences of each subtype may differ substantially [53]. Without distinguishing these subtypes, important variations in how specific visual processes contribute to reading challenges may have been overlooked, potentially limiting the precision of the findings and their broader theoretical interpretation [5]. Therefore, future research should consider phonological, linguistic, cognitive and attentional abilities to achieve a more nuanced understanding of the underlying mechanisms.

A further limitation of this study is related to the generalisation of the results. This investigation was conducted with Catalan-speaking children, a language spoken by approximately 10–12 million people [54], primarily located in Catalonia, Valencia, the Balearic Islands and Andorra. Catalan, a Romance language closely related to both Spanish and Occitan, is characterised by orthographic transparency and specific phoneme-grapheme correspondences, which may facilitate reading acquisition differently than in languages with less transparent orthographies, such as English. Consequently, the patterns observed in visual perception and reading performance among Catalan-speaking children may not generalise to populations speaking languages with substantially different orthographic or phonological properties. Future studies should investigate whether comparable associations between visual-perceptual skills and reading performance are observed in languages with more complex presenting characteristic orthographies, such as Chinese. Additionally, cross-national differences in educational systems and instruction may influence the development of visual-perceptual skills relevant to reading [55] and the extent to which such skills contribute to reading fluency and accuracy.

## Conclusions

Lower percentiles in visual discrimination, visual memory and form constancy, as assessed with the TVPS-3, were observed in children with reading disabilities, along with lower performance in overall reading, reading similar words and reading in different fonts. However, this study found no associations between visual perception abilities such as visual discrimination, visual constancy and visual memory for reading similar words and reading different fonts between the groups. Therefore, in clinical practice, low scores on these TVPS-3 subtests cannot be interpreted as contributing directly to a struggle to read similar words and difficulties reading in different fonts. Future research incorporating phonological, cognitive and language factors is needed to explore these relationships further.

## Supplementary Information

Supplementary Information

## Data Availability

No datasets were generated or analysed during the current study.
