# Supplementary material for: The Role of Visual Perception in Reading Across Fonts and Similar Words in Children with Reading Disabilities
Source: Ophthalmic Physiol Opt. 2026 May 6;46(3):560–7. doi: 10.1007/s44402-026-00094-4 (PMC13368950; doi:10.1007/s44402-026-00094-4)

**SUPPLEMENTARY INFORMATION**

**Table S1.** Word list of words in different fonts. The fonts are the following: Monotype italics, Lucida Console, Bradley Hand, Mistral, Harrington, and Calibri. For each font, there are 8 words.


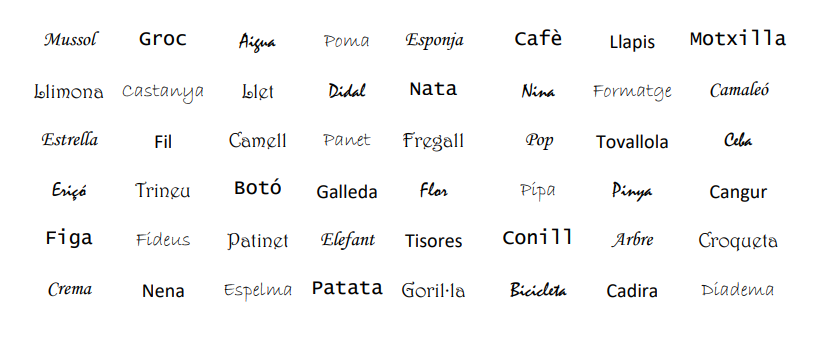


**Table S2.** Examples of font names, type of Serif (Yes, No), Stroke contrast (Low, Moderate, and High), and Crowding (Low, Normal, and High). Serifs are terminal extensions added to the ends of primary letter strokes. Stroke contrast refers to the intra-letter variation in stroke thickness within a typeface. Crowding* in typography is related to inter-letter spacing: as spacing decreases, crowding increases.

| Font Name | Example | Serif | Stroke contrast | Crowding |
| --- | --- | --- | --- | --- |
| Monotype Italics | Monotype Italics | Yes | Moderate | Normal |
| Lucida Console | Lucida Console | No | Low | Normal |
| Bradley Hand | Bradley Hand | No | Low | Normal |
| Mistral | Mistral | No | High | High |
| Harrington | Harrington | Yes | High | Normal |
| Calibri | Calibri | No | Low | Normal |

*In other areas, crowding is also referred as letter spacing and kerning, which describes the distances between letter pairs [1, pag 21]

[1]. Marshall, C. (2009). Reading and writing the electronic book. Morgan & Claypool Publishers.

**Figure S1.** Visual examples illustrating serif features, stroke contrast, and letter crowding in different font styles.


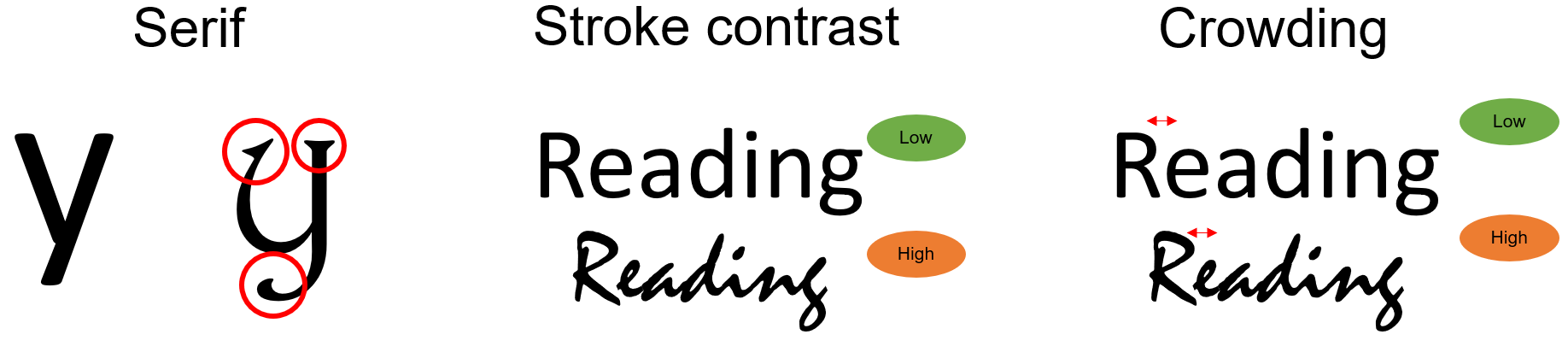


**Table S3.** Word list of similar words. Two lists of similar words, one of the one-syllables (on the top) for children under 9 years of age, and the other of two-syllables (on the bottom) for children aged 9 or older.

LIST OF ONE-SYLLABLES WORDS


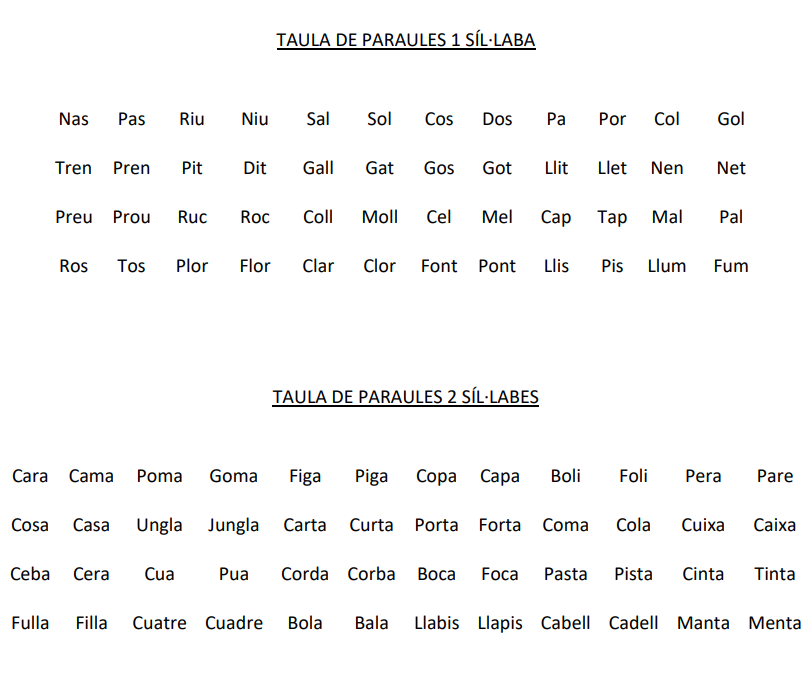


LIST OF TWO-SYLLABLES WORDS


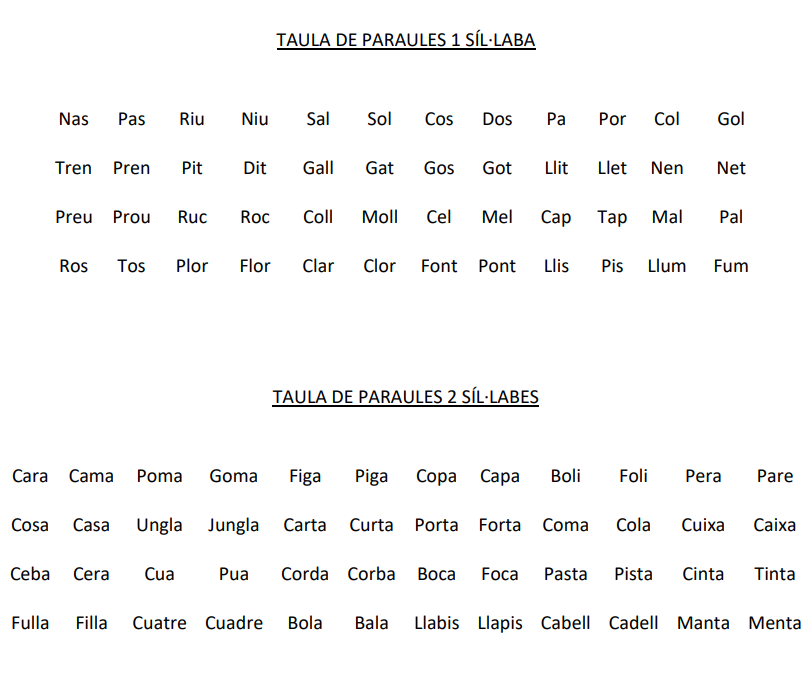

Supplement: Supplementary file 1 — Supplementary Information [file 44402_2026_94_MOESM1_ESM.docx]
